# Evidence of Sympatry of Clade A and Clade B Head Lice in a Pre-Columbian Chilean Mummy from Camarones

**DOI:** 10.1371/journal.pone.0076818

**Published:** 2013-10-30

**Authors:** Amina Boutellis, Rezak Drali, Mario A. Rivera, Kosta Y. Mumcuoglu, Didier Raoult

**Affiliations:** 1 Unité de Recherche sur les Maladies Infectieuses et Tropicales Emergentes: URMITE, Aix Marseille Université, UMR CNRS 7278, IRD 198, INSERM 1095. Faculté de Médecine, 27 Bd Jean Moulin, Marseille, France; 2 Programa Identidad del Fin del Mundo. Universidad de Magallanes-Mineduc, Punta Arenas, Chile; 3 Department of Microbiology and Molecular Genetics, The Kuvin Center for the Study of Infectious and Tropical Diseases, Hadassah Medical School, The Hebrew University, Jerusalem, Israel; Bangor University, United Kingdom

## Abstract

Three different lineages of head lice are known to parasitize humans. Clade A, which is currently worldwide in distribution, was previously demonstrated to be present in the Americas before the time of Columbus. The two other types of head lice are geographically restricted to America and Australia for clade B and to Africa and Asia for clade C. In this study, we tested two operculated nits from a 4,000-year-old Chilean mummy of Camarones for the presence of the partial *Cytb* mitochondrial gene (270 bp). Our finding shows that clade B head lice were present in America before the arrival of the European colonists.

## Introduction

*Pediculus humanus capitis* is an ancient human parasite most likely associated with humans since our pre-hominid ancestor and dispersed throughout the world by early human migrants [1]. Louse infestation in ancient human populations has been recorded in different geographic regions of the world [2] and even affected wealthy social classes, such as the 15^th^-century King of Naples, Ferdinand II of Aragon [3]. The oldest head louse nit was found on a hair from an archaeological site in northeastern Brazil and was dated to 8,000 B.C. [4]. The oldest such finding in Asia is 9,000 years old, obtained from a hair sample from an individual who lived in the Nahal Hemar cave in Israel [5]. Head lice have also been found at archaeological sites in the southwestern USA, the Aleutian Islands, Peru, Greenland and Mexico and on mummies that were Incan sacrifices [4]. Recently, another discovery of lice was reported for a Maitas Chiribaya mummy from Arica, in northern Chile, dating to 670–990 A.D. (calibrated) [6]. The evidence for the presence of ectoparasites on ancient Americans indicates that head lice most likely arrived with the first human colonists who entered the Americas [7]. However, by amplifying mitochondrial DNA (mtDNA) of part of two genes (*Cytb* and *Cox1*) belonging to 10,000-year-old lice collected from Peruvian mummies, Raoult and colleagues demonstrated that the worldwide clade A louse was in the Americas before the time of Columbus [8]. Two other clades of head lice have been reported and have a specific geographical distribution: clade C is specifically restricted to head lice in Ethiopia, Nepal and Senegal [9], whereas the clade B head lice are found in North and Central America, Australia and certain European countries [7]. However, the origin of clade B head lice remains unknown because no lice with this phylotype have been reported in Asia or in any region suspected to have contributed to populating the Americas. In the current study, two operculated nits from a mummy found in Camarones, Chile, were tested to identify the mitochondrial phylotypes of the lice.

## Materials and Methods

**Figure 1:**
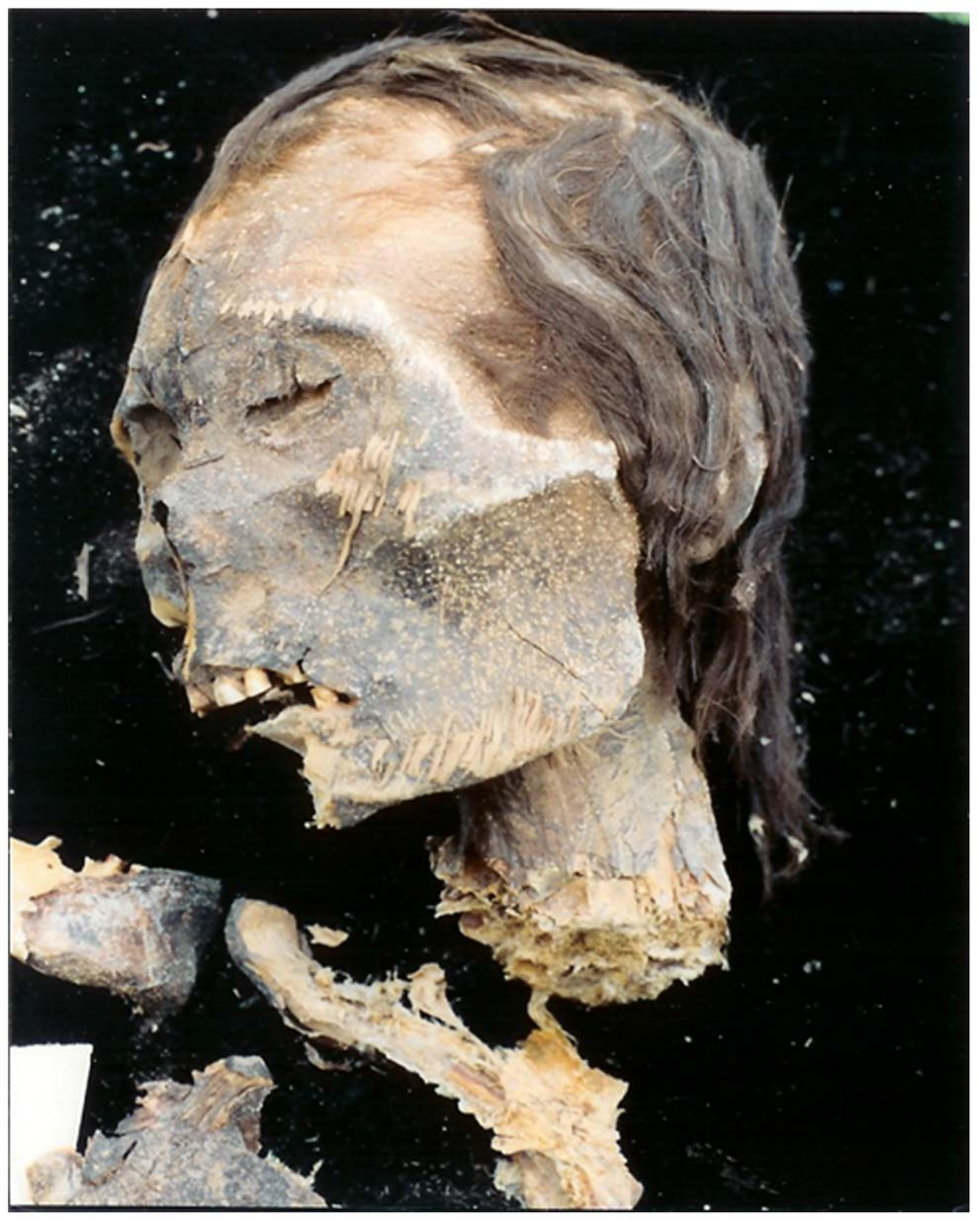
Picture of the Mummy. Mummy 23 from Camarones 15-D, Northern Chile © Mario A Rivera.

Hair samples from seven mummies from Camarones 15-D, Chile, carbon-dated to ca. 4,000 B.C., were examined for the presence of head lice (Figure 1), and in six hair samples, the nits of head lice were found. No body louse was found on these mummies. The material (nits fixed on hair) of one mummy was stored in 70% ethyl alcohol for the last 10 years and was sent to our laboratory in Marseilles in September 2012.

**Figure 2:**
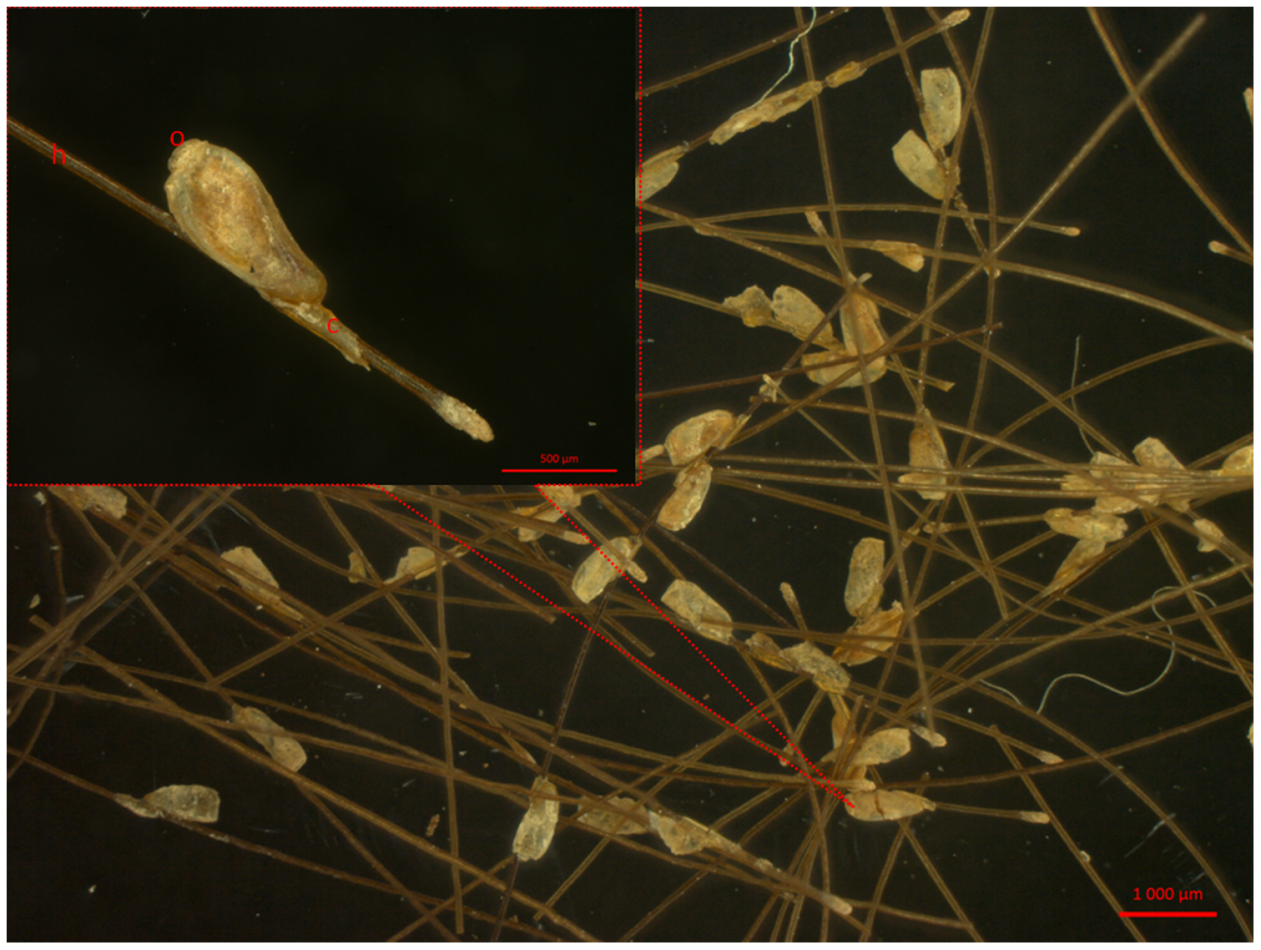
Picture of the Pre Columbian nit. A pre-Columbian nit isolated from a Chilean mummy (No. 1) (h: hair; o: operculum; c: cementum) (picture taken with a ZEISS AXIO ZOOM.V16).

A first screening of the quality of the nits was performed using our ZEISS zoom microscope with a fixed camera (ZEISS AXIO ZOOM.V16. France), and then two operculated nits were selected for our study (Figure 2). The two nits were rinsed twice in sterile water then crushed with a scalpel. The total genomic DNA was extracted and eluted in a 50 µl volume with a QIAamp Tissue Kit (Qiagen, Courtaboeuf, France) with an EZ1 apparatus, as described by the manufacturer. The extracted genomic DNA concentrations were 1.8 ng/µl and 1.3 ng/µl for the two nits, and the DNA samples were stored at −20°C under sterile conditions to avoid cross-contamination until further processing. The DNA of the two nits was amplified using a suicide nested polymerase chain reaction (PCR) protocol (re-amplification without positive control) with a partial *Cytb* gene (270 bp) primers, as previously described [11]. The same primers were used for both amplifications. PCR reactions were prepared on ice and contained 3 μl of the DNA template, 4 μl of 5X HF Phusion Buffer, 250 μM of each nucleotide, 0.5 μM of each primer, 0.2 μl of Phusion DNA Polymerase (Thermo Scientific, Lithuania) and water (DNase and RNase-Free) to a final reaction mixture volume of 20 μl. The PCRs were performed in a PTC-200 automated thermal cycler (MJ research, Waltham, MA, USA). The cycling conditions were 98°C for 30 sec; 40 cycles of 5 sec at 98°C, 30 sec at 56°C, 15 sec at 72°C; and a final extension time of 5 min at 72°C. All of the experiments were performed in a location free of louse DNA, under a hood with air capture and with sterilized instruments that were used only once. The negative controls remained negative. The success of the PCR amplification was then verified by migration of the PCR product on a 2% agarose gel. The NucleoFast 96 PCR Plates (Macherey-Nagel EURL, France) and BigDye Terminator version 1.1 cycle sequencing-ready reaction mix (Applied Biosystems, Foster City, CA) were then used to purify the PCR products to be sequenced directly in both directions with the same primers used in the PCR amplification. The ABI 3100 automated sequencer (Applied Biosystems) resolved the sequenced products. The program Chromas Pro software (Technelysium PTY, Australia) was used to analyze, assemble and correct the sequences.

In addition to our newly obtained data, 18 samples belonging to the worldwide clade A [8], 19 samples belonging to clade B from USA, UK [11] and Honduras [7] and 28 samples belonging to clade C from Senegal [9] and Ethiopia [12] were used.

The Phylogeny Reconstruction was performed from the DNA sequences using the Maximum Likelihood (ML) with 100 Bootstrap Replications within the MEGA 5 software with complete deletion, Tamura-Nei model (nucleotide) of substitution model was used automatically [13].

### Ethics statement

Mummies from Camarones were excavated in 1990 by a team of investigators under the direction of Mario A. Rivera, they have permit from Consejos Monumentos Nacionales, authorization number 355, November 12th, 1987. Samples were donated by Museo Arqueologia San Miguel Azapa, Universidad de Tarapaca, Arica, Chile and permission was obtained from the said Museum to access the collections [10].

## Results

**Figure 3:**
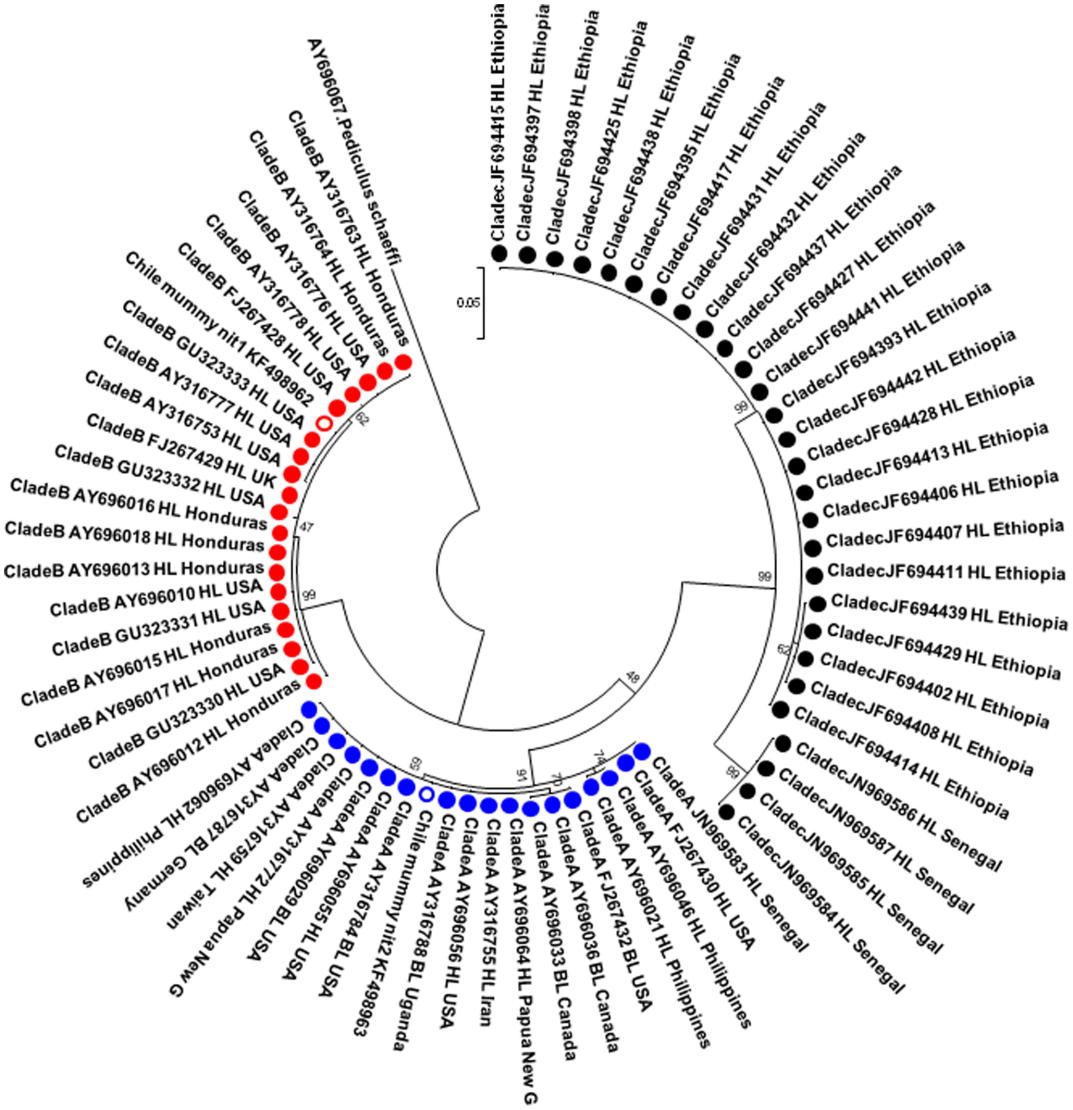
*Cytb* phylogenic analysis. The phylogenic tree based on ML method of the two pre-Columbian Chilean nits based on the partial *Cytb* gene (270 bp). **HL: head louse, BL: body louse.** The numbers on the branches are bootstrap values.

**Figure 4:**
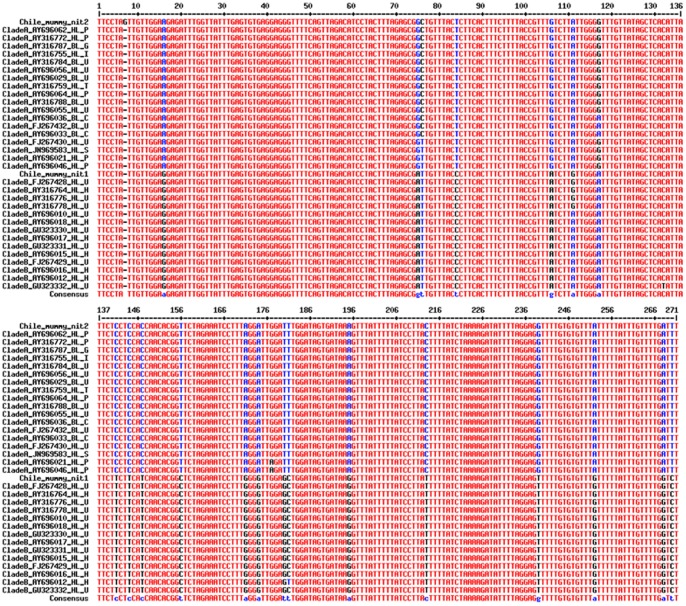
Sequences alignment. Alignment of the clade A and clade B sequences (Chilean mummy's nit and other sequences present in GenBank) P: Philippines, G: Germany, I: Iran, U: United State of America, T: Taiwan, C: Canada, S: Senegal, Pa: Papua New Guinea, H: Honduras.

The DNA of the two nits (848 µm and 912 µm long, respectively) was amplified and sequenced for the *Cytb* gene of 270 bp. After assembling the sequences and analyzing the phylogenetic tree, it was found that one nit belonged to the worldwide clade A (Genbank accession n°KF498963), whereas the second nit belonged to the clade B (Genbank accession n°KF498962) (figure 3). One base distinguished chilean mummy clade A head lice from other sequences found in GenBank: position 7 (G in mummy nit lice versus a gap in others) (Figure 4). There is no difference between the Chilean mummy clade B nit and other sequences present in GenBank (Figure 4).

## Discussion

We report the first identification of both the clade A and the clade B genotypes existing in sympatry in two nits isolated from human remains from pre-Columbian Chile. Lice with clade A and clade B mtDNA are not uncommon and were reported to be present on a single human head in both the USA and Honduras on several occasions [8]. Clade B was first identified in America and the United Kingdom and was later found in other European countries and Australia [7]. Until now, the origin of clade B was unknown, but according to the clade's current distribution, it was speculated that this louse phylotype was imported into Europe by Europeans returning from America [1]. Currently, the most likely theory is that the clade A louse issued from Africa and was distributed worldwide, given the clade's three different chromosomal signatures: A1, which is found worldwide; A2, which has been reported only in Africa; and A3, which is specific to American lice [14]. Clade B may have developed in North and Central America before Columbus and is now spreading throughout the world. This clade's origin predates modern *Homo sapiens* by an order of magnitude (ca. 1.18 million years) [1]. In contrast, Clade C is mostly confined to Africa and Asia.

The present work confirms that the origin of clade B was America before the arrival of Columbus, but it will be interesting to test other mummies from Asia, which is reputed to have peopled the Americas.
